# Urea cycle disorders in Argentine patients: clinical presentation, biochemical and genetic findings

**DOI:** 10.1186/s13023-019-1177-3

**Published:** 2019-08-19

**Authors:** Silene M. Silvera-Ruiz, José A. Arranz, Johannes Häberle, Celia J. Angaroni, Miriam Bezard, Norberto Guelbert, Adriana Becerra, Fernanda Peralta, Raquel Dodelson de Kremer, Laura E. Laróvere

**Affiliations:** 1 Centro de Estudio de las Metabolopatías Congénitas, Hospital de Niños de la Santísima Trinidad, Cátedra de Clínica Pediátrica, Fac. Cs. Médicas, UNC, Ferroviarios 1250, CP 5014 Córdoba, Argentina; 2 Unitat Metab, Hospital Vall d’Hebron, Barcelona, Spain; 3 University Children’s Hospital and Children’s Research Center, Zurich, Switzerland; 4 Sección Enfermedades Metabólicas, Hospital de Niños de la Santísima Trinidad, Córdoba, Argentina

**Keywords:** Urea cycle defects, Hyperammonemia, Ornithine transcarbamylase deficiency, Argininosuccinate synthetase deficiency, Argininosuccinate lyase deficiency

## Abstract

**Background:**

The incidence, prevalence, and molecular epidemiology of urea cycle disorders (UCDs) in Argentina remain underexplored. The present study is the first to thoroughly assess the clinical and molecular profiles of UCD patients examined at a single reference center in Argentina.

**Results:**

Forty-nine UCD cases were collected. About half (26/49, 53%) manifested neonatally with classical presentation and had a high mortality (25/26, 96%). Ornithine transcarbamylase deficiency (OTCD) was the most common UCD (26 patients). Argininosuccinate synthetase deficiency (ASSD) was detected in 19 cases, while argininosuccinate lyase deficiency (ASLD) was diagnosed in 4 cases. Molecular genetic analysis revealed 8 private *OTC* mutations and two large deletion/duplication events in the *OTC* gene. Most mutations in the *ASS1* and *ASL* genes were recurrent missense changes, and four alterations were novel. The clinical outcome of our UCD cohort was poor, with an overall mortality of 57% (28/49 cases), and a 28% (6/21) disability rate among the survivors.

**Conclusions:**

Most patients in our case series showed severe neonatal onset, with high morbidity/mortality. We detected in total 19 mutations, most of them recurrent and of high frequency worldwide. Noteworthy, we highlight the presence of a geographic cluster with high prevalence of a point mutation in the *ASS1* gene. This study suggests that these disorders may be more frequent than commonly assumed, and stresses the need for increased awareness amongst health professionals and greater availability of diagnostic tools for accurate identification, early diagnosis, and timely treatment.

**Electronic supplementary material:**

The online version of this article (10.1186/s13023-019-1177-3) contains supplementary material, which is available to authorized users.

## Synopsis

This is the first case series of urea cycle deficiency patients from Argentina diagnosed at a single center, describing a high prevalence of neonatal onset, and confirming high recurrence of common worldwide mutations plus some private mutations first described in our cohort.

## Introduction

The urea cycle is the final common pathway for the excretion of waste nitrogen as well as arginine synthesis [1]. Urea cycle disorders (UCDs) are inborn errors of ammonia detoxification/arginine synthesis caused by mutations in one of five core enzymes, one activating enzyme, or one of two mitochondrial antiporters. Enzymatic defects include N-acetylglutamate synthase deficiency (NAGSD; MIM#237310), carbamoylphosphate synthetase 1 deficiency (CPS1D; MIM#237300), ornithine transcarbamylase deficiency (OTCD; MIM#311250), argininosuccinate synthetase deficiency (ASSD; MIM#215700), argininosuccinate lyase deficiency (ASLD; MIM#207900), and arginase 1 deficiency (ARG1D; MIM#207800). Two transporters are involved in the cycle, the ornithine/citrulline antiporter (ORNT1), associated with the hyperornithinemia-hyperammonemia-homocitrullinuria (HHH) syndrome (MIM #238970), and the glutamate/aspartate antiporter (CITRIN), whose deficiency gives rise to citrullinemia type 2 (MIM #605814 and #603471). All these deficiencies are inherited in an autosomal recessive manner, except for OTCD which has X-linked recessive inheritance, and in some cases arises from spontaneous mutations in germ cells.

The incidence of UCDs has recently been determined to be 1 in 35,000 births, although it varies between populations. About two-thirds of all UCDs are due to mutations in OTC, while mutations in ASS1 and ASL account for, one-fifth and one-tenth of cases, respectively. However, the overall incidence might be higher because not all cases are detected, and underdiagnosis of fatal cases is common [2].

The onset and severity of UCDs are highly variable and depend both on the specific mutation involved and its impact on the corresponding enzymatic or transport function. The onset of severe forms usually occurs during the neonatal period and is characterized by food refusal, vomiting, lethargy, polypnea, and rapid progression to coma and multiorgan failure due to hyperammonemia [3, 4]. The onset of mild forms can occur at any age, with hyperammonemic episodes triggered during catabolic stress (infections, vomiting, surgery, etc.) or with more insidious symptoms such as failure to thrive, chronic liver disease, developmental delay, behavioral disorders, and psychiatric symptoms [5]. Delayed diagnosis, often as a result of lack of symptom awareness amongst families and primary health care physicians, results in either death or cognitive impairment [6].

The Argentinean population is a unique mix of several ethnicities, with expected low consanguinity. So far, only a few UCDs case reports originated from Argentina [7–9]. The present study summarizes our experience regarding diagnosis, genetic testing, and outcomes of 49 UCD patients from 36 families evaluated at a single referral center in Argentina.

## Materials and methods

### UCD diagnosis

Clinical symptoms of UCD patients included lethargy, lack of appetite, persistent vomiting, intractable seizures, unexplained neurological alterations, neurodegeneration, developmental delay, coma, unexplained recurrent liver dysfunction, and cholestasis. Samples from subjects with clinical suspicion of UCD underwent biochemical and genetic analyses in our laboratory.

### Biochemical analyses

A key biomarker for UCDs is hyperammonemia (> 100 μM; > 1 y old) in the absence of high anion gap and with a normal plasma glucose level. Laboratory findings characteristic of UCDs include elevated levels of plasma glutamine and alanine, and high or low plasma concentrations of citrulline, arginine and argininosuccinic, wich allow to determine the enzymatic block of the urea cycle [4]. Determination of plasma and urinary amino acids was done using HPLC according to the technique of Duran et al. (2008) [10]. Quantification of plasma ammonia was done by enzymatic spectrophotometric assay (Randox Ammonium Kit, Randox Laboratories LTD, UK). Measurement of orotic acid in urine by HPLC was performed according to the technique of Simmonds et al. (1991, [11]).

### Molecular analyses

All subjects, or their parents or legal guardians, provided consent for DNA testing. Extraction of genomic DNA was carried out using the purification protocol of the Wizard Genomic DNA Promega Purification Kit (Promega, Madison, USA). Genetic analyses consisted of amplification of the gene/exon of interest from genomic DNA with specific oligonucleotides by PCR, and examination of the sequence using either restriction enzymes or by direct Sanger sequencing (ABI 3130XL automatic capillary sequencer, Applied Biosystems). If more extensive gene analysis was needed (i.e. for large deletions, duplications, or for complete sequencing of exons and intronic regions) we used Single Strand Conformational Polymorphism or Multiplex Ligation dependent Probe Amplification.

## Results

### Patient series data

**Fig. 1:**
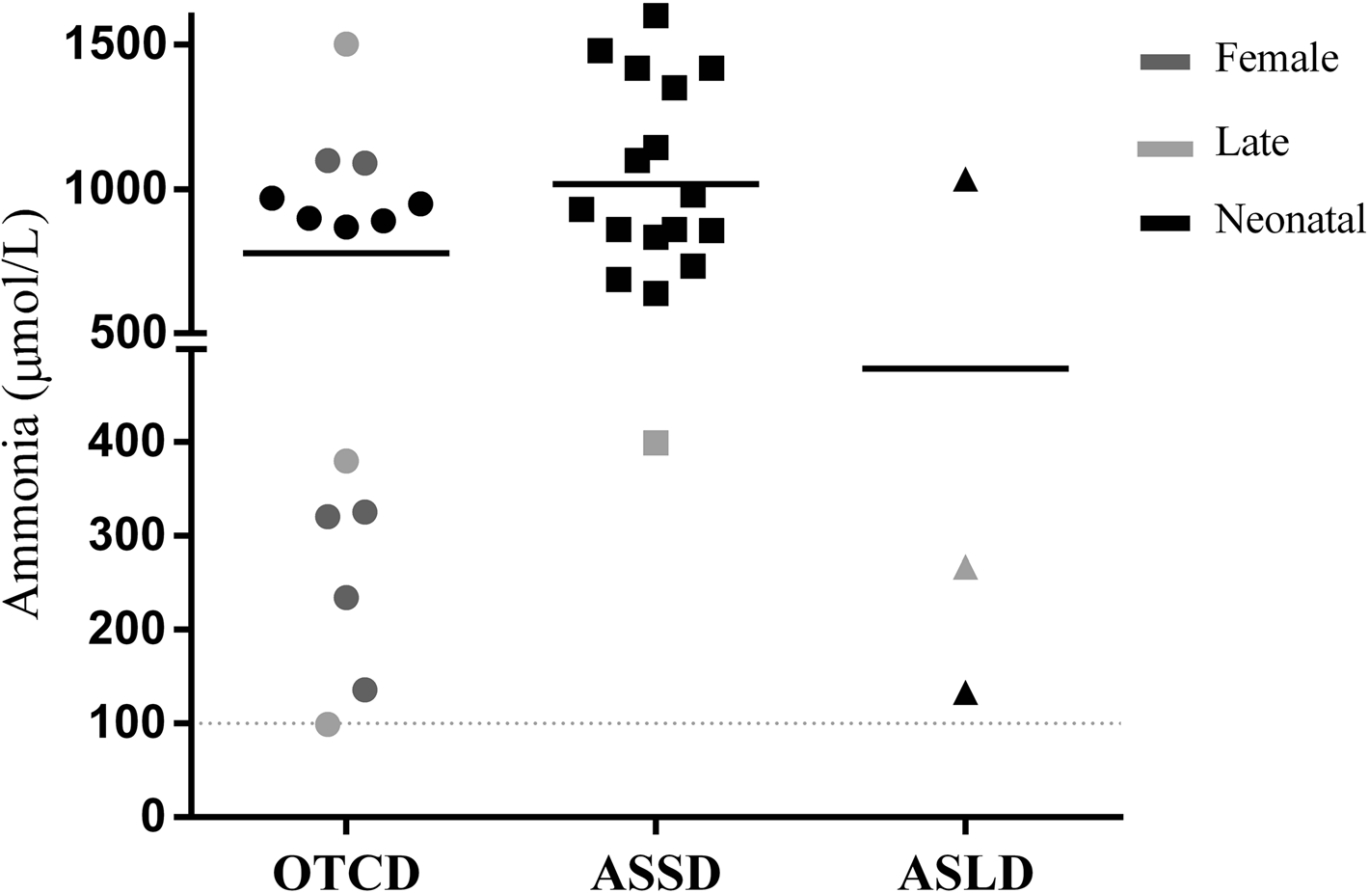
Plasma ammonia levels at UCDs diagnosis. Grey dotted lines indicate normal plasma ammonia (bottom: upper limit in children > 1 year old) and recommended value for hemodialysis (top; [22]). Black solid lines indicate mean values for each UCD

**Fig. 2:**
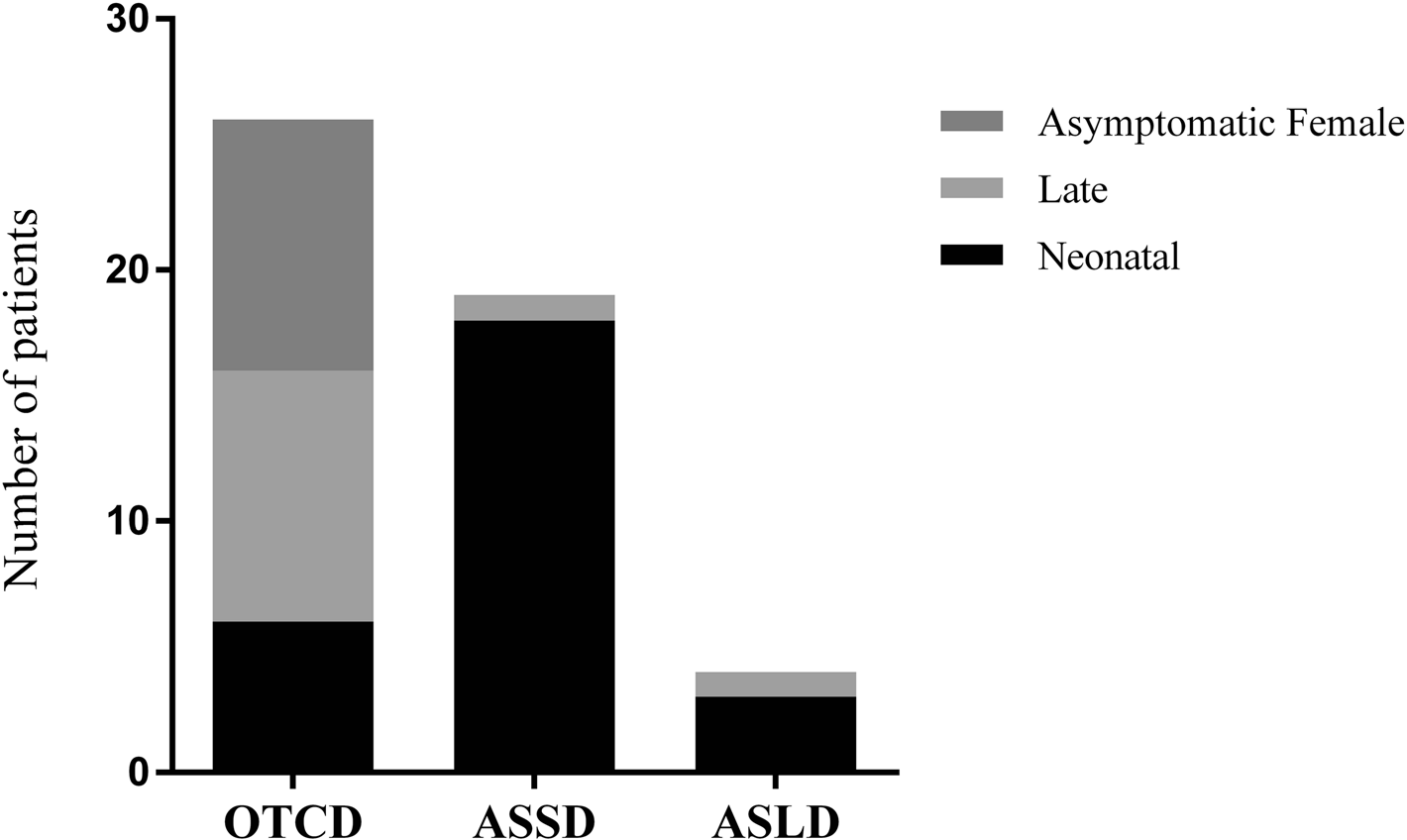
UCDs’ onset. Grayscale stacked bars indicate the number of cases with neonatal, late-onset, or asymptomatic presentation in each disease. OTCD asymptomatic females are shown as a separate group and described in the text

Table 1 lists UCDs patient data from our reference center. It includes 49 cases and their corresponding clinical presentation, genetic findings, and disease onset times. OTCD was the most common UCD, observed in 26/49 patients, followed by ASSD, detected in 19 patients, and by ASLD, diagnosed in 4 patients. About half of the cases (26/49, 53%) presented in the neonatal period with classical presentation, and had a high mortality (25/26, 96%) (Additional file 1: Table S1). Plasma ammonia values for all 49 patients are shown in Fig. 1. Clinical manifestations varied, leading to high mortality among boys diagnosed in the neonatal period or with late-onset UCD forms, and included also asymptomatic and severely affected women. Symptoms began in the neonatal period in 55% (5/9) of cases in hemizygous males; neonatal forms were not observed in symptomatic carriers, but late-onset disease was present in 43% (7/17) of the female cases (Fig. 2).

**Table 1:** CEMECO’s UCDs cohort description

Fam	Pat	Presentation	Gene	Alteration	Protein	Onset	Remarks
I	1	Symptomatic female	*OTC*	delExon1–10		7 m	ND †
	2	Symptomatic female	*OTC*	delExon 1–10		3y	ND, DA
	3	Hemizygous neonatal onset	*OTC*	delExon 1–10		5d	ND †
	4	Asymptomatic female	*OTC*	delExon1–10			
	5	Asymptomatic female	*OTC*	delExon1–10			
II	6	Hemizygous late onset	*OTC*	c.216 + 1G > A	Affects splicing	2y	
	7	Asymptomatic female	*OTC*	c.216 + 1G > A	Affects splicing		
III	8	Hemizygous late onset	*OTC*	c.386G > A	Affects splicing	6 m	ND
	9	Asymptomatic female	*OTC*	c.386G > A	Affects splicing		
IV	10	Symptomatic female	*OTC*	c.452 T > G	p.Leu151Arg	4y	ND
V	11	Symptomatic female	*OTC*	c.533C > T	p.Thr178Met	10 m	ND
	12	Hemizygous neonatal onset	*OTC*	c.533C > T	p.Thr178Met	48-72hs	ND †
	13	Asymptomatic female	*OTC*	c.533C > T	p.Thr178Met		
VI	14	Symptomatic female	*OTC*	c.540 + 1G > A	Intronic	9 m	ND
VII	15	Hemizygous neonatal onset	*OTC*	c.540 + 1G > A	Intronic	48-72hs	
	16	Asymptomatic female	*OTC*	c.540 + 1G > A	Intronic		
VIII	17	Hemizygous late onset	*OTC*	c.622G > A	p.Ala208Thr	8y	
	18	Asymptomatic female	*OTC*	c.622G > A	p.Ala208Thr		
IX	19	Hemizygous late onset	*OTC*	c.829C > T	p.Arg277Trp	10y	†
	20	Asymptomatic female	*OTC*	c.829C > T	p.Arg277Trp		
X	21	Symptomatic female	*OTC*	dup1–9/del10	–	9 m	†
XI	22	Hemizygous neonatal onset	*OTC*	c.697delG	p.Ala233Glnfs*14	48-72hs	†
	23	Asymptomatic female	*OTC*	c.697delG	p.Ala233Glnfs*14		
	24	Asymptomatic female	*OTC*	c.697delG	p.Ala233Glnfs*14		
XII	25	Hemizygous neonatal onset	*OTC*	NA	NA	48-72hs	†
XIII	26	Symptomatic female	*OTC*	NA	NA	48-72hs	†
XIV	27	Late	*ASS1*	c.79 T > C / c.847G > A	p.Gln27* / p.Glu283Lys	45d	
XV	28	Late	*ASS1*	c.79 T > C / c.970G > A	p.Gln27* / p.Gly324Ser	16d	†
XVI	29	Neonatal	*ASS1*	c.1168G > A /c.1168G > A	p.Gly390Arg /p.Gly390Arg	48-72hs	†
XVII	30	Neonatal	*ASS1*	c.1168G > A /c.1168G > A	p.Gly390Arg /p.Gly390Arg	48-72hs	†
XVIII	31	Neonatal	*ASS1*	c.1168G > A /c.1168G > A	p.Gly390Arg /p.Gly390Arg	48-72hs	†
XIX	32	Neonatal	*ASS1*	c.1168G > A /c.1168G > A	p.Gly390Arg /p.Gly390Arg	48-72hs	†
XX	33	Neonatal	*ASS1*	c.1168G > A /c.1168G > A	p.Gly390Arg /p.Gly390Arg	48-72hs	†
XXI	34	Neonatal	*ASS1*	c.1168G > A /c.1168G > A	p.Gly390Arg /p.Gly390Arg	48-72hs	†
XXII	35	Neonatal	*ASS1*	c.1168G > A /c.1168G > A	p.Gly390Arg /p.Gly390Arg	48-72hs	†
XXIII	36	Neonatal	*ASS1*	c.1168G > A /c.1168G > A	p.Gly390Arg /p.Gly390Arg	48-72hs	†
XXIV	37	Neonatal	*ASS1*	c.1168G > A /c.1168G > A	p.Gly390Arg /p.Gly390Arg	48-72hs	†
XXV	38	Neonatal	*ASS1*	c.1168G > A /c.1168G > A	p.Gly390Arg /p.Gly390Arg	48-72hs	†
XXVI	39	Neonatal	*ASS1*	c.1168G > A /c.1168G > A	p.Gly390Arg /p.Gly390Arg	48-72hs	†
XXVII	40	Neonatal	*ASS1*	c.1168G > A /c.1168G > A	p.Gly390Arg /p.Gly390Arg	48-72hs	†
XXVIII	41	Neonatal	*ASS1*	c.1168G > A /c.1168G > A	p.Gly390Arg /p.Gly390Arg	48-72hs	†
XXIX	42	Neonatal	*ASS1*	c.1168G > A /c.1168G > A	p.Gly390Arg /p.Gly390Arg	48-72hs	†
XXX	43	Neonatal	*ASS1*	c.1168G > A /c.1168G > A	p.Gly390Arg /p.Gly390Arg	48-72hs	†
XXXI	44	Neonatal	*ASS1*	c.1168G > A /c.1168G > A	p.Gly390Arg /p.Gly390Arg	48-72hs	†
XXXII	45	Neonatal	*ASS1*	c.1168G > A /c.1168G > A	p.Gly390Arg /p.Gly390Arg	48-72hs	†
XXXIII	46	Neonatal	*ASL*	c.857A > G/c.328G > T	p.Gln286Arg/p.Gly110*	48-72hs	†
XXXIV	47	Late	*ASL*	c.857A > G/c.436C > T	p.Gln286Arg/p.Arg146Trp	20 m	
XXXV	48	Neonatal	*ASL*	c.857A > G/c.857A > G	p.Gln286Arg/p.Gln286Arg	48-72hs	ND
XXXVI	49	Neonatal	*ASL*	c.857A > G/c.857A > G	p.Gln286Arg/p.Gln286Arg	48-72hs	†

*ND* Neurologic damage, †: deceased; *DA* Diagnosis in asymptomatic period, *NA* Not assessed

Besides hyperammonemia as main diagnostic biomarker, the patient series presented high levels of urinary orotic acid (average 1733 μmol/mmol creatinine, range 117–6879 μmol/mmol creatinine, NV < 10 μmol/mmol creatinine [10]). This confirmed that the enzyme block was downstream of CPS1, thus ruling out possible CPS1D or NAGSD.

### Ornithine transcarbamylase deficiency

A total of 26 patients belonging to 12 families were diagnosed with OTCD; 17/26 were women and 9/26 were men. The mean age of onset of symptoms was 29.9 months (48 h-10 years). Whereas neurological damage was present in most cases, disease manifestations sometimes differed among patients with the same mutation.

For OTCD patients mean ammonia level at the beginning of the symptoms was 778.1 μmol/L (range: 98–2181; Fig. 1), mean glutamine levels were also elevated (2065 μmol/L; range: 277–4229), while citrulline and arginine values were within normal range (Table 2).

**Table 2:** Mean values and ranges (μmol/L) of plasma metabolites in UCD patients

Disease	Ammonia	Glutamine	Citrulline	Arginine	Argininosuccinate
Control	< 100	333–809	16–32	44–120	0–0.7
OTCD	778.1 (98–2181)	2065.44 (277–4229)	14.6 (0–28)	66.0 (8–135)	–
ASSD	1017.1 (399–1600)	2863.24 (1123-4504)	3203.8 (758–8500)	12.9 (8–25.2)	–
ASLD	478.3 (133–1035)	1346.32 (374–2913)	222.3 (75–357)	10.7 (4–23)	80.7 (37–179)

Control: Reference values and ranges for pediatric population (> 1 year old) [10]

We were able to determine the disease-causing mutation in all but two patients, as we lost contact with their parents and could not obtain the respective DNA samples. The alterations found in the other 24 patients, and their clinical presentations, are listed in Table 1. These included: 4 hemizygotes with neonatal onset (complete *OTC* gene deletion, c.533C > T, c.540 + 1G > A, c.697delG); 4 hemizygotes with late onset (c.216 + 1G > A, c.386G > A, c.622G > A, c.829C > T); 6 symptomatic heterozygotes (complete *OTC* gene deletion, c.533C > T, c.452 T > G, c.540 + 1G > A, dupE1–9/delE10); and 10 asymptomatic females with an almost complete representation of the mutational spectrum within this cohort, implying a more favorable lyonization in these patients.

### Argininosuccinate synthetase deficiency

Nineteen patients belonging to 19 families were identified with ASSD (11 females, 57.9%; 8 males, 42.1%) (Table 1). Neonatal forms were detected in all but two cases.

Severe disease manifestation, characterized by symptoms in the first hours of life and death in the neonatal period, predominated in this cohort. The two other cases, detected at 45 and 16 days of life, progressed with severe hyperammonemic crisis and metabolic decompensation (Fig. 2). Cognitive and developmental deterioration could be observed in one of these patients (patient 28), who died at 18 months.

Mean ammonia level at the onset of ASSD symptoms was 1017 μmol/L (range: 399–1600; Fig. 1), while mean glutamine level (2863 μmol/L; range: 1123-4504; Table 2) was higher than in OTCD due to the large number of severe neonatal cases of ASSD. Characteristically, ASSD patients had highly increased plasma citrulline (3203 μmol/L; range: 758–8500), while arginine levels were below the normal range (12.9 μmol/L; range: 8.0–25.2) (Table 2).

Exact determination of the *ASS1* genotype could be achieved in all patients, in many cases deductively from the DNA of parents heterozygous for the molecular defect. Thus, genetic screening of the 19 affected families allowed us to identify the alterations described in Table 1. Among those families, 17 presented the same mutation in homozygosity, namely c.1168G > A (p.Gly390Arg), and 2 nonrelated families carried the same recurrent mutation, c.79 T > C (p.Gln27*), in heterozygosity. The other two mutations found in these compound heterozygotes were two previously reported missense changes: c.847G > A (p.Glu283Lys) and c.970G > A (p.Gly324Ser) [12, 13].

### Argininosuccinate lyase deficiency

Four patients from 4 families (1 woman and 3 males) were identified with ASLD (Table 1). The mean age at onset of symptoms was 2 days in the 3 neonatal forms, with two deaths registered in the neonatal period, and 20 months in the late-onset form. The latter patient showed no severe hyperammonemic crisis or metabolic decompensation, and the main clinical features were hypotonia and trichorrhexis nodosa with baldness periods. Cognitive and developmental deterioration could be observed in one of the patients with neonatal onset (patient 48).

For all ASLD patients average ammonia level at the onset of symptoms was 478 μmol/L (range: 133–1035; Fig. 1). Glutamine was also elevated (1346 μmol/L; range: 374–2913; Table 2). Argininosuccinate levels were, as expected for ASLD, extremely high (80 μmol/L; range: 37–179), while arginine levels were within normal range (Table 2).

Molecular assays on the *ASL* gene allowed us to determine the specific causal mutations and establish accurate diagnoses. The mutations found were c.328G > T (p.Gly110*), c.436C > T (p.Arg146Trp), and c.857A > G (p.Gln286Arg), all previously reported in the literature [14, 15].

## Discussion

This study reports the first comprehensive case series of UCDs in Argentina. Using the latest estimated incidence of UCDs of 1 in 35,000 [2], and a birth rate of 457,335 live births per year in Argentina (Censo Nacional de Población, Hogares y Viviendas 2010), an average of 13 new UCDs patients can be expected each year in Argentina. Summar et al. (2013) [2] reported that 26% of patients were symptomatic in the newborn period and 69% of all patients had symptoms at some point. This should result in a minimum of 9 UCDs patients with hyperammonemia per year in Argentina, 4 of them presenting neonatal onset. However, the average incidence calculated from national registries is 4 new UCDs patients per year. We speculate that more patients may be diagnosed at other clinics and medical centers in Argentina and not being reported, but is also likely that many cases are not being correctly identified.

### Onset and follow up

We found several precipitating factors of hyperammonemic crises in our UCD patient cohort, among them abandonment of diet and/or pharmacological treatment, and infectious processes; these increased morbidity markers, hospitalization rates and lengths of stay, and led to a greater use of ammonium chelators. The mortality rate in our cohort (53%) is higher than reported for similar case series. A possible explanation may be the high prevalence of neonatal onset citrullinemia in a circumscribed area, added to underreporting of mild cases in our population. Notwithstanding, the data from this study confirm that neonatal onset UCD patients are at high risk for not only initial life-threatening decompensation but also for recurrent hyperammonemic crises and peak ammonia values, which may result in permanent neurological damage [16]. Of note, the presence of high ammonia levels in symptomatic females or late-onset forms, indicates that late forms are not always mild. Since the severity of the crisis is multifactorial, phenotype prediction is important to prevent and effectively manage future crises.

Treatment and follow-up of UCD symptomatic patients in our center follows international guidelines with respect to diet, supplements, and medication [3]. Additional adverse factors in developing countries like Argentina are inadequate facilities in most primary and secondary level hospitals that contribute to poor outcomes. For instance, laboratory ammonia assays are usually available only in tertiary hospitals. Therefore, patients may die without a diagnosis, or the disease is diagnosed late, contributing to increased morbidity and mortality. Identification of UCDs in the affected families allowed accurate retrospective diagnoses and medical advice to parents regarding future pregnancies. Meanwhile, early diagnosis meant an integral improvement in the quality of life of patients, by implementing timely and adequate treatment and follow-ups.

### Mutation Spectrum

Our previous report assessed three unreported OTCD-causing mutations: c.540 + 1G > A, c.697delG, and dup1–9/del10, and highlighted the relevance of combining molecular and bioinformatics analyses for accurate diagnosis and outcome prediction in patients with suspected OTCD [9]. We matched clinical, biochemical, and molecular findings with bioinformatics analyses to report genotype-phenotype correlations in this OTCD case series [9].

We found four *ASS1* mutations in our population: c.79 T > C (p.Gln27*), c.847G > A (p.Glu283Lys), c.970G > A (p.Gly324Ser) and c.1168G > A (p.Gly390Arg). The mutation c.79 T > C (p.Gln27*) was first described in our population and blocks the enzymatic activity of ASS by producing a stop codon in exon 3; this variant was recently found associated with severe neonatal onset in an Arab homozygous patient [17]. It should be noted that two patients in our group presented this mutation although there is no relationship between these patients, nor known consanguinity between parents. The c.847G > A (p.Glu283Lys) mutation described by Gao et al. (2003) [13] in a homozygous patient produces a severe phenotype. The coexistence of this mutation with the c.79 T > C mutated allele in patient 27 generates a significant structural change in the enzyme, explaining the clinical presentation of the child, i.e. protein intolerance and frequent metabolic decompensation despite treatment with high doses of sodium phenylbutyrate. The missense mutation c.970G > A (p.Gly324Ser) described by Kobayashi et al. (1990) [14] has been identified exclusively in patients with a severe phenotype. The G324 residue is strictly conserved in all reported homologous sequences of ASS. This substitution breaks the helical structure of α-helix 10, preventing the binding of citrulline and aspartate [17]. In vitro bacterial expression systems used to validate this mutation show a null ASS activity [18]. It is speculated that the coexistence of these two mutated alleles (c.79 T > C / c.970G > A) in patient 28 generates a significant structural change in the enzyme. This is expected to aggravate clinical evolution, but besides protein intolerance and significant psychomotor-cognitive delay, there are rare metabolic decompensations under treatment with sodium benzoate and good management of plasma ammonia levels. At 18 months of age this patient had a hyperammonemic crisis (peak ammonia = 480 μmol/L) concurrent with hypotonia, epilepsy, vomiting, and respiratory distress, and died after 5 days of hospitalization despite intensive care measures.

The c.1168G > A (p.Gly390Arg) mutation found in the majority of patients of this cohort was previously described by Engel et al. (2009) [19] with high prevalence worldwide. This alteration represented 88.8% of all the mutated *ASS1* alleles in our ASSD cohort, a rate higher than the 27–62.5% described globally by Diez-Fernandez (2017) [16]; the carrier frequency of p.Gly390Arg is 4.1% or 1/25 inhabitants; however, the incidence of ASSD in our center, of 1 in 2427 children [7], is approximately twenty times higher than the worldwide incidence of 1 in 57,000 reported by Brusilow and Horwich (2001) [1], and for this reason this substitution was specifically assessed by our group [7]. Genealogy analysis of several affected families suggests a transmission ratio distortion of the mutated allele compared to the expected frequency [20, 21]; thus, occurrence of the disease in descendants of couples at risk is 57.89%, i.e. more than twice the frequency expected for an autosomal recessive disease. Due to the high incidence of ASSD in our country, we proposed that preconceptional diagnosis of carriers is the most rational preventive measure for the management of ASSD, for which there is still no effective treatment [7].

The mutations in the *ASL* gene were c.328G > T (p.Gly110*), c.436C > T (p.Arg146Trp), and c.857A > G (p.Gln286Arg), all previously reported in the literature [14, 15]. The mutation p.Gln286Arg variant has a high frequency worldwide and was the subject of many studies for its capacity for intragenic complementation. All the alterations found are severe, causing lack of ASL activity, but due to the intragenic complementation phenomenon, the patient that is compound heterozygous for c.857A > G/c.436C > T (patient 47) had late-onset and presented minimal manifestations of the deficiency (Table 1).

## Conclusion

This is the first comprehensive report of mutations in UCDs from Argentina. However, because our study is restricted to patients diagnosed in a single reference center, it may not be representative of the overall incidence of UCDs in the country. To improve the prognosis of these patients it would be of great importance to expand neonatal screening for UCDs, incorporating newer diagnostic and therapeutic tools, and to create a national UCD registry to know the true incidence of these diseases. Likewise, educating pediatricians, neurologists, and neonatologists about UCDs and their symptoms will allow to diagnose more patients at a presymptomatic state or when ammonia levels are still below the threshold that causes irreversible neurological damage.

## Additional file

Additional file 1: **Table S1.** UCDs in Argentina: Onset and outcome. (DOCX 15 kb)

## Data Availability

All data generated or analyzed during this study are included in this published article [and its supplementary information files].

## Abbreviations

ASLD: Argininosuccinate lyase deficiency
ASSD: Argininosuccinate synthetase deficiency
OTCD: Ornithine transcarbamylase deficiency
UCD: Urea cycle disorder
