# Supplementary material for: Urea cycle disorders in Argentine patients: clinical presentation, biochemical and genetic findings
Source: Orphanet J Rare Dis. 2019 Aug 19;14:203. doi: 10.1186/s13023-019-1177-3 (PMC6700778; doi:10.1186/s13023-019-1177-3)
Supplement: Supplementary file 1 — Table S1. UCDs in Argentina: Onset and outcome. (DOCX 15 kb) [file 13023_2019_1177_MOESM1_ESM.docx]

**Additional file 1: Table S1.** UCDs in Argentina: Onset and outcome.

| UCD | OTCD | ASSD | ASLD | Total |
| --- | --- | --- | --- | --- |
| Total number | 26 | 19 | 4 | 49 |
| Onset age |  |  |  |  |
| Neonatal | 6 | 17 | 3 | 26 |
| Infancy | 5 | 0 | 0 | 5 |
| > Infancy (> 1 year) | 5 | 2 | 1 | 8 |
| Clinical presentation |  |  |  |  |
| Neonatal or intermittent encephalopathy, or seizures | 6 | 17 | 3 | 26 |
| Liver disease | 8 | 18 | 3 | 29 |
| Failure to thrive | 0 | 0 | 1 | 1 |
| Developmental delay | 7 | 1 | 1 | 9 |
| Other neuropsychiatric/ behavioural/ ataxia/ spasticity symptoms | 6 | 1 | 1 | 8 |
| Outcome |  |  |  |  |
| Death | 8 | 18 | 3 | 29 |
| Alive | 18 | 1 | 1 | 20 |
| Development: Delayed | 7 | 1 | 1 | 9 |
| Development: Normal or near normal | 11 | 0 | 0 | 11 |
| Diet (Protein restriction) + medications | 7 | 1 | 1 | 9 |
|  |  |  |  |  |
